# Thiopyrano[2,3-*d*]Thiazoles as New Efficient Scaffolds in Medicinal Chemistry

**DOI:** 10.3390/scipharm86020026

**Published:** 2018-06-14

**Authors:** Anna Kryshchyshyn, Olexandra Roman, Andrii Lozynskyi, Roman Lesyk

**Affiliations:** 1 Department of Pharmaceutical, Organic and Bioorganic Chemistry, Danylo Halytsky Lviv National Medical University, Pekarska 69, 79010 Lviv, Ukraine; kryshchyshyn.a@gmail.com (A.K.); lozisnskij@i.ua (A.L.); 2 Department of General, Inorganic and Bioorganic Chemistry, Danylo Halytsky Lviv National Medical University, Pekarska 69, 79010 Lviv, Ukraine; lesia_roman@ukr.net

**Keywords:** 4-thiazolidinones, thiopyrano[2,3-*d*]thiazoles, [4+2]-cycloaddition, biological activity

## Abstract

This review presents the up to date development of fused thiopyranothiazoles that comprise one of the thiazolidine derivatives classes. Thiazolidine and thiazolidinone-related compounds belong to the widely studied heterocycles from a medicinal chemistry perspective. From the chemical point of view, they are perfect heterodienes to undergo *hetero*-Diels–Alder reaction with a variety of dienophiles, yielding regio- and diastereoselectively thiopyranothiazole scaffolds. The annealing of thiazole and thiopyran cycles in condensed heterosystem is a precondition for the “centers conservative” creation of the ligand-target binding complex and can promote a potential selectivity to biotargets. The review covers possible therapeutic applications of thiopyrano[2,3-*d*]thiazoles, such as anti-inflammatory, antibacterial, anticancer as well as aniparasitic activities. Thus, thiopyrano[2,3-*d*]thiazoles may be used as powerful tools in the development of biologically active agents and drug-like molecules.

## 1. Introduction

**Figure 1:**
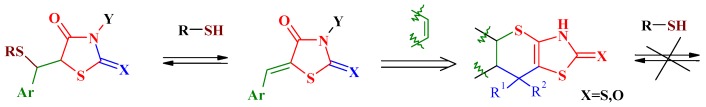
Background for thiopyrano[2,3-*d*]thiazoles design.

The thiopyranothiazole scaffold is characterized by “fixed” 4-thiazolidinone biophor in a “rigid” condensed system that allows to save the biological activity of their synthetic precursors 5-ene-4-thiazolidinones. 4-Thiazolidinones and related heterocycles comprise a sufficiently studied class of compounds that reveal a wide spectrum of biological activities, such as anti-inflammatory, antimicrobial, antifungal, antiviral, anticancer, anticonvulsant, antituberculous [1,2,3,4], etc. Among thiazolidinone derivatives, there are a number of drug candidates as well as approved drugs, for example, anti-hyperglycemic glitazones (PPARγ agonists)—Roziglitazone, Pioglitazone (2,4-thiazolidinedione derivatives) [5]; aldose reductase inhibitor—Epalrestat (rhodanine derivative) [6]; anti-inflammatory drug—double cyclooxygenase-2/5-lipooxygenase inhibitor—Darbufelon (2-amino-4-thiazolidone derivative) [7]; diuretic Ethozoline (2-ene-4-thiazolidinone derivative) [8]; anticonvulsant Ralitolin [9]. However, recently, such a rich pharmacological profile of thiazolidinones is considered by some medicinal chemists not as an advantage, but as a drawback. For example, 5-ene-4-thiazolidinones, being the most active subgroup, is classified as PAINS (pan assay interference compounds) that are defined by their ability to show activity across a range of assays and towards a range of proteins [10,11]. This is argued by a variety of inherent biological activity, low selectivity and the ability of 5-ylidene-4-thiazolidinones to be Michael acceptors. Despite this, rigorous selection based on SAR analysis and proved selectivity leave such compounds a «right to life» in medicinal chemistry [12,13,14]. Moreover, the objects of our review (thiopyranothiazoles) can be considered as bio-mimetics of pharmacologically active 5-ene-4-thiazolidinones without the mentioned Michael acceptors properties (Figure 1) [15,16,17]. The combination of thiazole and thiopyran cycles in condensed heterosystem is a precondition for the creation of “centers conservative” of the ligand-target binding complex and promotes potential selectivity to biotargets. Considering the mentioned arguments, the directed search for new chemotherapeutic agents among thiopyrano[2,3-*d*]thiazole derivatives is a justified and promising direction in modern medicinal chemistry. In this review, we tried to systematize the data on chemistry and pharmacology of thiopyrano[2,3-*d*]thiazoles from the perspective of medicinal and pharmaceutical chemistry.

## 2. *Hetero*-Diels-Alder Reaction as a Key Approach for the Synthesis of Thiopyrano[2,3-*d*]Thiazole Derivatives

**Figure 2:**
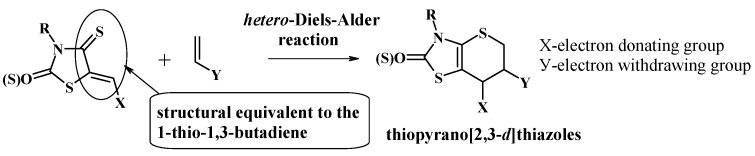
5-arylidene-4-thiazolidinethiones as heterodienes.

The most effective approach to thiopyrano[2,3-*d*]thiazole system design is the use of the *hetero*-Diels–Alder reaction. Mentioned approach has been described for the first time by I.D. Komaritsa [18], N.A. Kassab et al. [19,20] who had successfully used 5-arylidene-4-thioxo-2-thiazolidinones (5-arylideneisorhodanines) and 5-arylidene-2,4-thiazolidinedithiones (5-arylidenethiorhodanines) as heterodienes. Mentioned reagents contain in their structure α,β-unsaturated thiocarbonyl fragment similar to the 1-thio-1,3-butadiene which leads to their high reactivity in the [4+2]-cycloaddition reactions (Figure 2). According to the molecular orbital theory, Diels–Alder reaction is based on the overlay of the diene’s “HOMO” and dienophile’s “LUMO”. The important condition for this reaction is the presence of strong dienophile with electron acceptor properties to decrease energy difference between diene’s “HOMO” and “LUMO” or “HOMO” of the dienophile. For these reasons reactions are highly regioselective and form products according to the molecular orbital theory.

### 2.1. Utilization of 5-Arylidene-4-Thiazolidinethiones in the Hetero-Diels–Alder Reactions

**Scheme 1:**
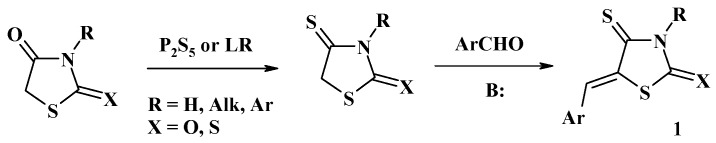
Synthesis of 5-arylidene-4-thiazolidinethiones.

5-arylideneiso- and thiorhodanines **1** are prepared in the Knoevenagel reaction of the corresponding 4-thiazolidinethiones [21] which in turn are synthesized in the thionation reaction of 2-thioxo-4-thiazolidinone (rhodanine) and 2,4-thiazolidinedione (Scheme 1). The thionation reaction of 4-thiazolidinones is commonly carried out in anhydrous dioxane with P_2_S_5_ [22,23] or Lawesson’s reagent (LR)—(2,4-bis(4-methoxyphenyl)-1,3-dithiadiphosphetane-2,4-dithione) [24]. A green method of 5-arylidene-4-thioxothiazolidines synthesis in PEG medium at room temperature without adding any catalyst has been recently reported [25].

**Scheme 2:**
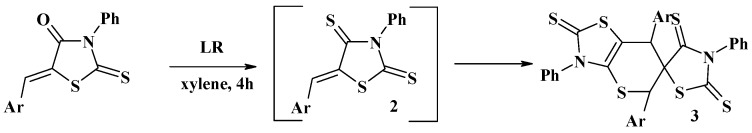
Thionation of 5-arylidene-3-phenylrhodanines.

It is important to note that thionation of 5-arylidene-3-phenylrhodanines with the Lawesson’s reagent in the xylene medium can result in the formation of thione’s dimers **3**, due to the spontaneous [4+2]-cycloaddition reaction of the intermediate 5-arylidene-2,4-thiazolidinedithiones **2** (Scheme 2). The mentioned synthesis leads to the formation of thiorhodanine spiro-substituted thiopyrano[2,3-*d*]thiazoles **3** with high yields and the absence of by-products [24].

**Scheme 3:**
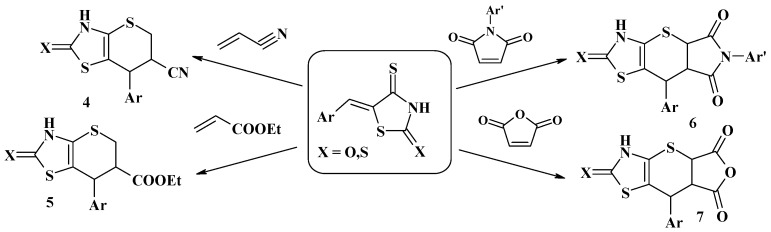
Pioneering works on chemistry of thiopyrano[2,3-*d*]thiazoles.

In pioneering works on chemistry of thiopyrano[2,3-*d*]thiazoles dienophile component was represented by maleic acid and its derivatives (maleic anhydride, maleinimides) and acrylic acid and its derivatives (methyl acrylate, ethyl acrylate, acrylonitrile), therefore allowing to obtain compounds **4–7** (Scheme 3) [18,19,20]. 

**Scheme 4:**
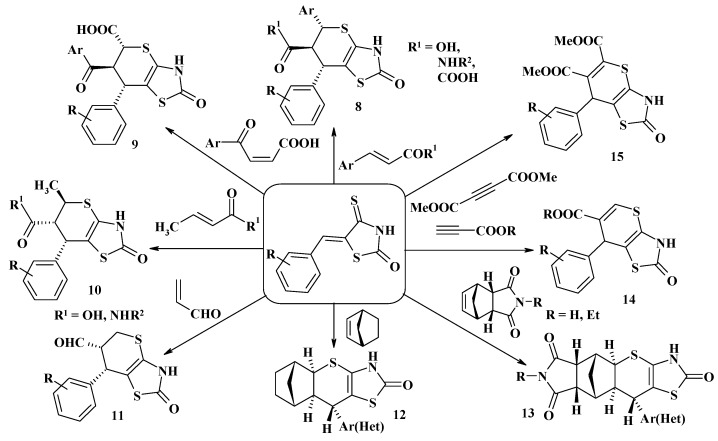
The synthesis of thiopyrano[2,3-*d*]thiazoles using cynnamic acids and their amides, aroylacrylic and arylidene pyruvic acids, dimethyl acetylenedicarboxylate, propiolic acid and its ethyl ester, acroleine, 2-norbornene and 5-norbornene-2,3-dicarboxylic acid imides as dienophiles.

Currently, the list of dienophiles for the synthesis of thiopyrano[2,3-*d*]thiazole derivatives has significantly expanded. Thus, the use of cynnamic acids [26] and their amides [27], aroylacrylic [28] and arylidene pyruvic [29] acids as well as dimethyl acetylenedicarboxylate [30], propiolic acid and its ethyl ester [26], acroleine [31], 2-norbornene [15] and 5-norbornene-2,3-dicarboxylic acid imides [16] as dienophiles allowed to obtain new thiopyrano[2,3-*d*]thiazoles **8–15** as promising biologically active compounds based on the “thiazolidinone” matrix (Scheme 4). It should be noted that the presence of chiral centers in the structure of thiopyrano[2,3-*d*]thiazole cycle causes certain features of stereochemistry in the *hetero*-Diels–Alder reaction. The given issue became the subject of an intense study considering the current trends in organic and medicinal chemistry. It was found that the above-mentioned [4+2]-cycloadditions are regio- and diastereoselective.

**Scheme 5:**
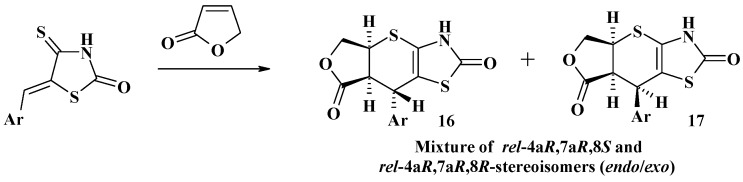
Features of the reaction of 5-arylidene-4-thioxo-2-thiazolidinones with 2(5*H*)furanone.

The reaction of 5-arylideneisorhodanines with 2(5*H*)furanone yields mixtures of endo/exo adducts **16**,**17**. (Scheme 5). Considering moderate diastereoselectivity of the process, the reaction can occur through endo or exo transition states resulting in different positions of the protons at C-8 of core heterocycle. Thus, the endo transition state leads to anti configuration, while the exo geometry results in syn configuration of the H-8 respectively. Endo and exo adducts can be separated by column chromatography [32].

**Scheme 6:**
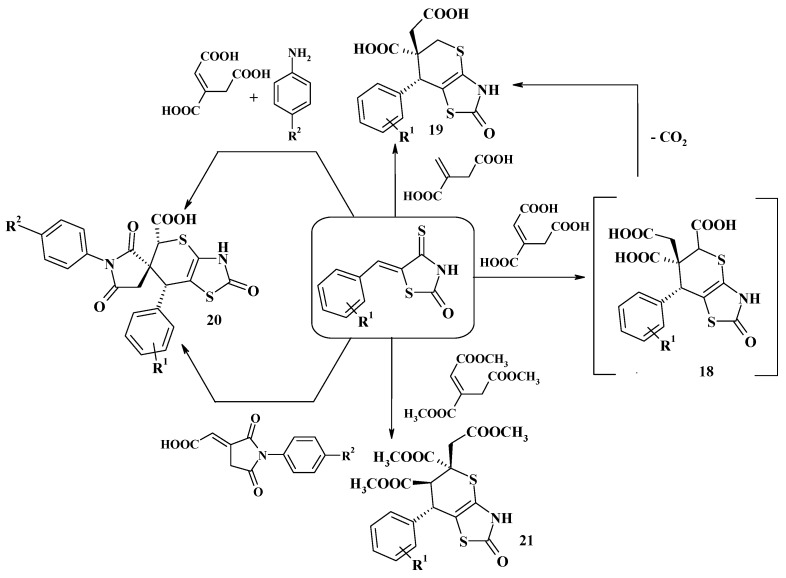
*Trans*-Aconitic acid motif in the synthesis of thiopyrano[2,3-*d*]thiazoles.

The reaction of 5-arylideneisorhodanines with *trans*-aconitic acid proceeds as a regio- and diastereoselective [4+2]-cycloaddition with spontaneous decarboxylation of the adduct **18** to furnish *rel-*(6*R*,7*R*)-diastereomers **19**. The same products were synthesized using itaconic acid as dienophile. Interestingly that one-pot three-component reaction of 5-arylideneisorhodanines, trans-aconitic acid and anilines diastereoselectively yielded *rel*-(5′*R*,6′*R*,7′*R*)-spiro[pyrrolidin-3,6′-thiopyrano[2,3-*d*]thiazol]-2,2′,5-triones **20** [33,34] without decarboxylation of adducts. The thiopyrano[2,3-*d*]thiazoles **20** were contrary synthesized using (2,5-dioxo-1-arylpyrrolidin-3-ylidene)-acetic acids as dienophiles. It should be noted that unlike free trans-aconitic acid or its imides, corresponding trimethyl ester (trimethyl 1-propene-1,2,3-tricarboxylate) reacted with 5-arylideneisorhodanines with opposite regioselectivity resulting [4+2]-cycloadducts (**21**) (Scheme 6) [33].

**Scheme 7:**
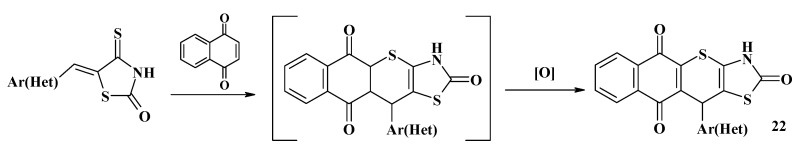
Synthesis of thiopyrano[2,3-*d*]thiazoles containing a naphthoquinone moiety.

In the case of 1,4-naphthoquinone utilization as dienophile intermediates of the [4+2]-cycloaddition reaction undergo spontaneous oxidation with the formation of tetracyclic thiopyrano[2,3-*d*]thiazoles **22** (Scheme 7) [17].

**Scheme 8:**
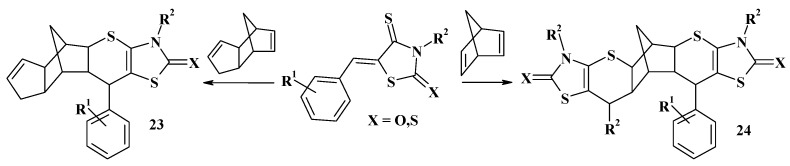
Synthesis of thiopyrano[2,3-*d*]thiazoles containing a norbornane moiety.

The compounds 5-arylidene-4-thiazolidinethiones with dicyclopentadiene and norbornadiene react in a similar manner as norbornene derivatives (Scheme 4, compounds **12**,**13**) [35]. In the case of the norbornadiene symmetrical bis-adduct **23** regioselectively forms. The structure of derivative **24** is predictable (Scheme 8). 

**Scheme 9:**
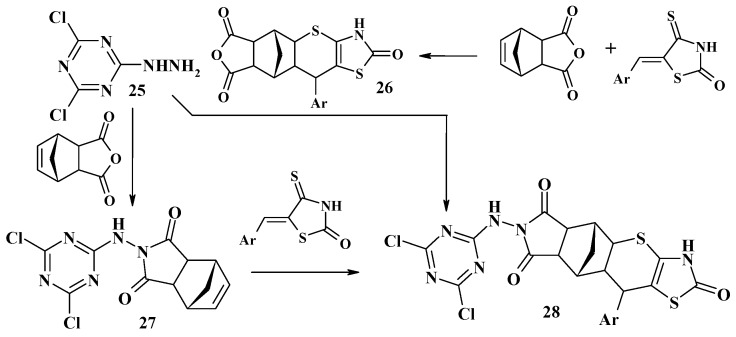
Diversification of thiopyranothiazoles bearing norbornane moiety.

For the diversification of thiopyranothiazoles bearing norbornane moiety (4,6-dichloro-1,3,5-triazin-2-yl)-hydrazine **25** was used to obtain fused thiopyrano[2,3-*d*]thiazoles **28** via two alternative methods (Scheme 9). One way involved obtaining compounds **26** in *hetero*-Diels-Alder reaction of 5-arylidene-4-thioxo-2-thiazolidinones with bicyclo[2.2.1]hept-5-ene-2,3-dicarboxylic anhydride (endic anhydride) followed by the condensation with triazinelhydrazine in toluene medium and presence of thionyl chloride. Whilst the other way includes condensation of triazine with endic anhydride in DMF medium at 110 °C firstly and then the reaction of 4-(4,6-dichloro-1,3,5-triazin-2-ylamino)-4-azatricyclo[5.2.1.0^2,6^]dec-8-ene-3,5-dione **27** with 5-arylidene-4-thioxo-2-thiazolidinones yielding corresponding [4+2]-cycloaddition products **28** [36].

**Scheme 10:**
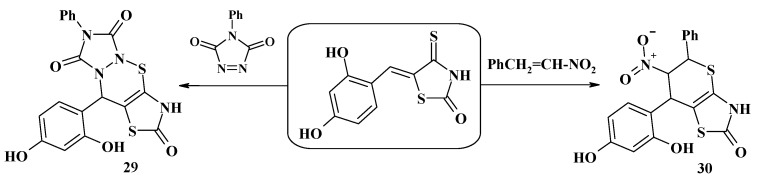
The synthesis of thiopyrano[2,3-*d*]thiazoles using *N*-phenyl-1,3,4-triazole-2,5-dione and ω-nitrostyrene as dienophiles.

*N*-Phenyl-1,3,4-triazole-2,5-dione and ω-nitrostyrene (Scheme 10) turned out to be effective dienophyles in the reaction with 5-(2,4-dihydroxybenzylidene)-4-thioxo-2-thiazolidinone that allowed obtaining 3,8-dihydro-1,4-dithia-3,4a,6,7a-tetraza-s-indacene **29** and 6-nitro-substituted thiopyrano[2,3-*d*]thiazole **30** [19,37]. 

**Scheme 11:**
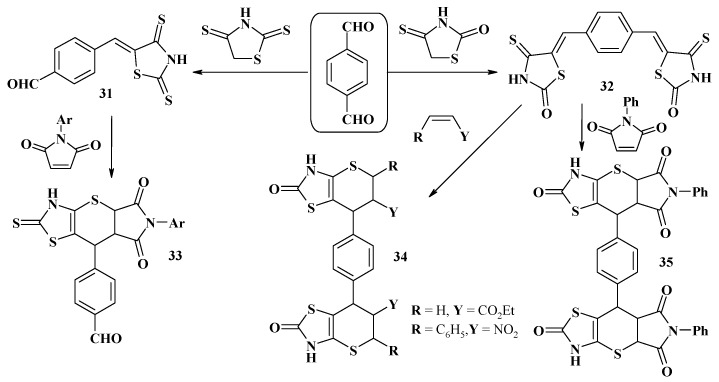
The synthesis of 1,4-bis(thiopyrano[2,3-*d*]thiazolyl)benzene derivatives.

N.H. Metwally and colleagues [38] had chosen terephthalic aldehyde, thio- and isorhodanines as starting reagents in order to investigate the peculiarities of the *hetero*-Diels–Alder reaction. On the basis of these starting reagents monoarylidene thiorhodanine **31** and bicyclic arylidene isorhodanine derivatives **32** were prepared in the Knoevenagel condensation and than used as heterodienes. Phenyl maleinimide, ethyl acrylate and ω-nitrostyrene were used as dienophiles that enabled syntheses of the compounds **33–35** (Scheme 11). 

### 2.2. Usage of 5-Alkylidene-4-Thiazolidinethiones in the Synthesis of Thiopyrano[2,3-d]Thiazole Core 

**Scheme 12:**
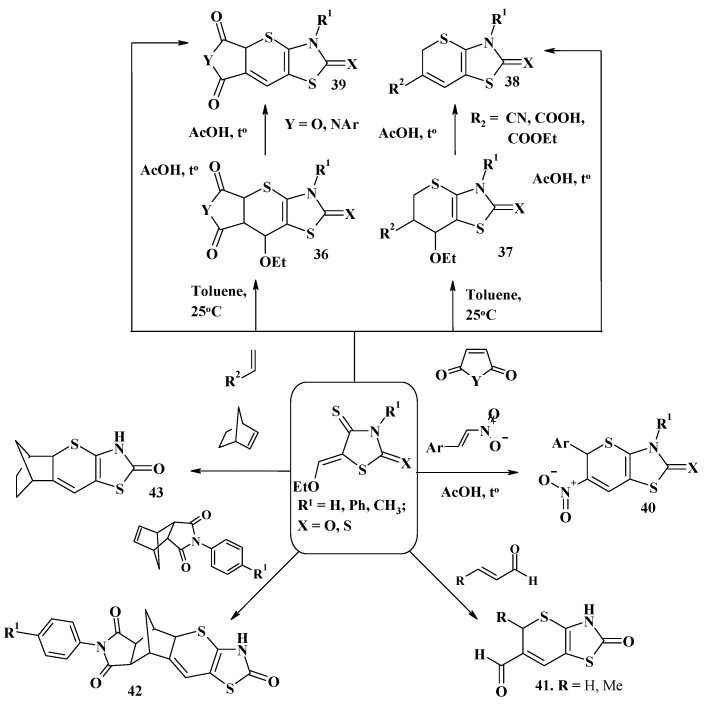
5-Ethoxymethylideneiso- and thiorhodanines as heterodienes in the synthesis of thiopyrano[2,3-*d*]thiazoles.

5-Alkylidene-4-thiazolidinediones are useful and important reagents in the search for biological active compounds, as they allow obtaining thiopyrano[2,3-*d*]thiazole derivatives with relatively low molecular weight. Among them 5-ethoxymethylideneisorhodanine and corresponding thiorhodanine are the most interesting and effective heterodienes that significantly expanded structural diversity of thiopyrano[2,3-*d*]thiazoles (Scheme 12). In the early works [18,39] dedicated to this issue a number of specific features of mentioned 4-thiazolidinethiones in *hetero*-Diels–Alder reaction were experimentally established and outlined. In particular, [4+2]-cycloaddition of 5-ethoxymethylideneiso- and thiorhodanines with appropriate dienophiles in the toluene medium at room temperature passes as a classical *hetero*-Diels–Alder reaction with the formation of 7-ethoxy-3,5,6,7-tetrahydro-2*H*-thiopyrano[2,3-*d*]thiazoles **36**,**37**. When changing the reaction medium to the acetic acid and heating the reaction mixture, elimination of the ethanol molecule with the formation of an additional double bond and the corresponding 3,5-dihydro-2*H*-thiopyrano[2,3-*d*]thiazoles **38**–**40** is observed. Analogous pattern was observed in the [4+2]-cycloaddition with acrolein, crotonic aldehyde, 2-norbornene and 5-norbornene-2,3-dicarboxylic acid imides in the medium of acetic acid that allowed obtaining heterocyclic aldehydes **41** and polycyclic heterosystems **42**,**43** [40]. 

**Scheme 13:**
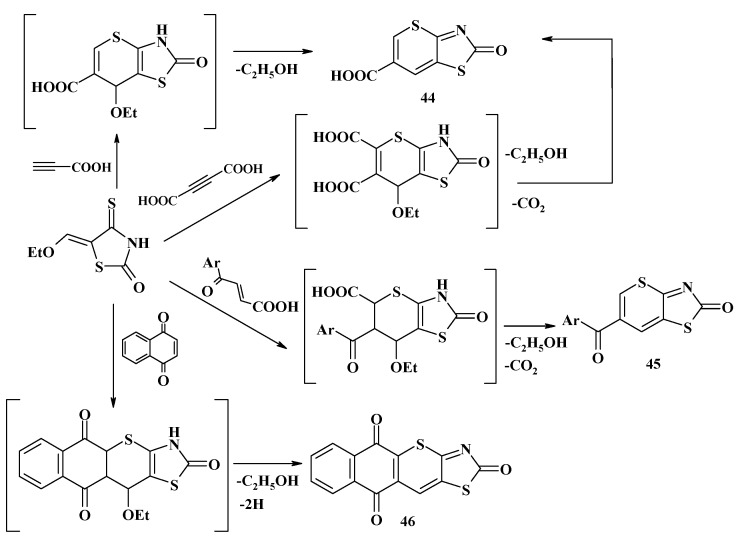
5-Ethoxymethylideneisorhodanine as versatile building block for novel biorelevant small molecules with thiopyrano[2,3-*d*]thiazole core.

Interaction of 5-ethoxymethylene-4-thioxo-2-thiazolidinones with propiolic acid is accompanied by not only the elimination of ethanol, but the rearrangement of double bonds with the formation of 2-oxo-2*H*-thiopyrano[2,3-*d*]thiazole-6-carboxylic acid **44**. The compound **44** is also formed when using acetylene dicarboxylic acid as dienophile that may be explained by the similar transformation of [4+2]-cycloadduct and additional decarboxylation in the 5th position. [4+2]-Adducts of aroylacrylic acids and 5-ethoxymethylideneisorhodanine also undergo elimination of ethanol and decarboxylation with regioselective formation of 6-aryl-2-oxo-3,5-dihydro-2*H*-thiopyrano[2,3-*d*]thiazolyl-6-methanones **45**. At the same time, interaction with the 1,4-naphthoquinone was accompanied by spontaneous intermediate dehydrogenation with the formation of additional endocyclic double bond and elimination of the ethanol molecule yielding 5,10-dihydro-2*H*-benzo[6,7]thiochromeno[2,3-*d*][1,3]thiazole-2,5,10-trione **46** (Scheme 13) [40]. 

**Scheme 14:**
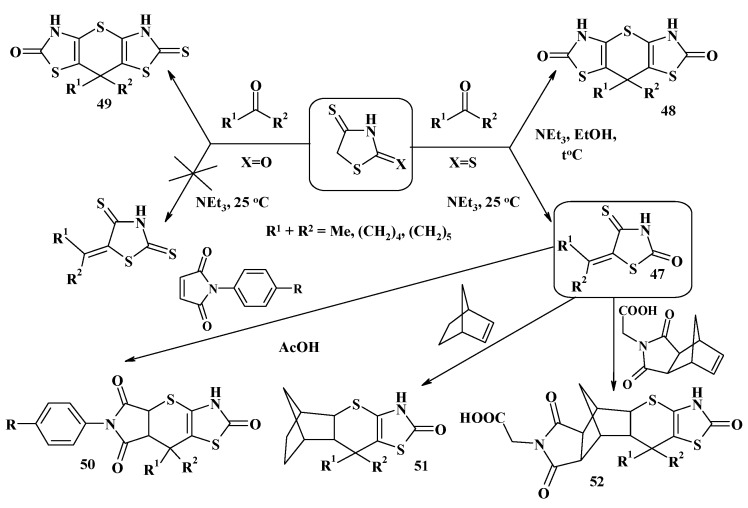
5-(Cyclo)alkylideneisorhodanine motif in the synthesis of thiopyrano[2,3-*d*]thiazoles.

One of the relatively new areas in thiopyrano[2,3-*d*]thiazole chemistry is the usage of 5-(cyclo)alkylideneisorhodanines as key reagents in [4+2]-cycloaddition (Scheme 14). Thus, the initial heterodienes **47** were obtained in the reaction of isorhodanine with acetone, cyclopentanone or cyclohexanone at room temperature and in the presence of triethylamine as catalyst. Interestingly, performing the reaction in ethanol medium at the solvent boiling point leads to the formation of tricyclic heterosystems **48**. When thiorhodanine is used, only condensed derivatives **49** are formed regardless of the reaction conditions. [4+2]-Cycloaddition of 5-(cyclo)alkylideneisorhodanines with arylmaleinamides, 2-norbornene and (3,5-dioxo-4-azatricyclo[5.2.1.0^2,6^]decen-8-yl-4)-acetic acid [36] yielded low molecular thiopyrano[2,3-*d*]thiazoles **50**–**52** [41].

## 3. The Michael Reaction and Related Processes in the Synthesis of Thiopyrano[2,3-*d*]Thiazoles

**Scheme 15:**
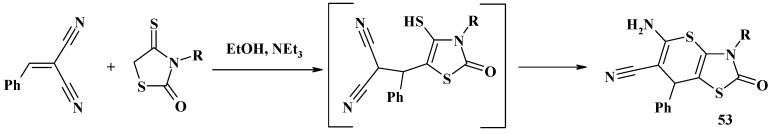
Activated nitriles in the synthesis of thiopyrano[2,3-*d*]thiazoles.

The Michael reaction is one more effective approach to the synthesis of thiopyrano[2,3-*d*]thiazoles (Scheme 15). Thus, the interaction of arylmethylene malononitrile and 3-substituted isorhodanines in the medium of absolute ethanol at the presence of triethylamine gave 5-amino-2-oxo-7-phenyl-3,5,6,7-tetrahydro-2*H*-thiopyrano[2,3-*d*]thiazole-6-carbonitriles **53** [42]. 

**Scheme 16:**
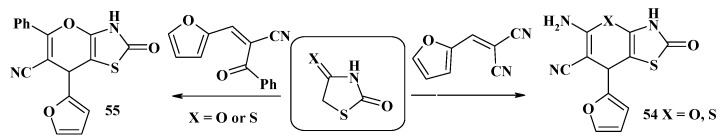
The reactions of α-substituted 2-(2-furyl)-acrylonitriles with isorhodanine and thiazolidinedione.

F.M. Abdelrazek and coauthors have used the above approach for the synthesis of pyrano[2,3-*d*]thiazoles and their thioanalogs (Scheme 16) [43]. The reaction of isorhodanine with 2-(furan-2-yl)-methylenemalononitrile yields thiopyrano[2,3-*d*]thiazole-6-carbonitrile **54** (X=S), and when 2,4-thiazolidinedione is used pyrano[2,3-*d*]thiazole **54** (X=O) is formed. Interestingly, 2-benzoyl-3-(furan-2-yl)-acrylonitrile in the Michael reaction with 2,4-thiazolidinedione or isorhodanine, at the elimination of water or hydrogen sulphide respectively forms the same compound—7-(furan-2-yl)-2-oxo-5-phenyl-3,7-dihydro-2*H*-pyrano[2,3-*d*]thiazole-6-carbonitrile **55**. 

**Scheme 17:**
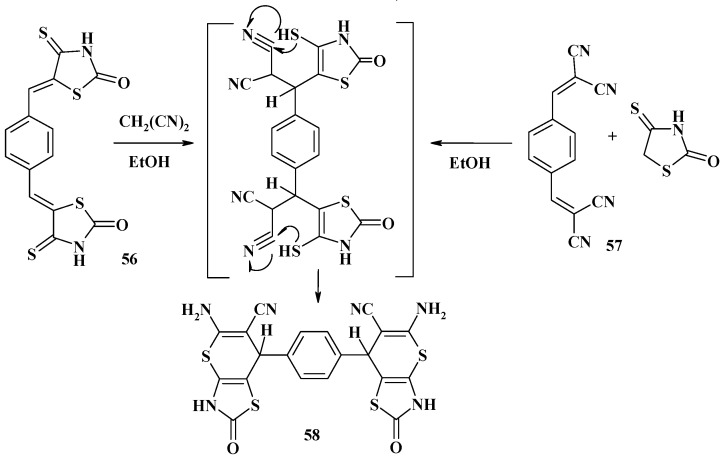
Synthesis of bis-thiopyrano[2,3-*d*]thiazole derivative in Michael addition reactions.

When studying the peculiarities of Michael addition of bicyclic 5-arylideneiso(thio)rhodanines **56** with malonodinitrile bis-thiopyrano[2,3-*d*]thiazole derivative **58** was obtained, which early was synthesized in the reaction of 1,4-bis-(2,2′-dicyanovinyl)-benzene **57** with two equivalents of isorhodanine. In the second case, formation of the derivative **58** occured as two-stage process including an initial Michael reaction with further cyclization of the intermediate by the attack of cyano group with mercapto group of thiazole cycle (Scheme 17) [38].

**Scheme 18:**
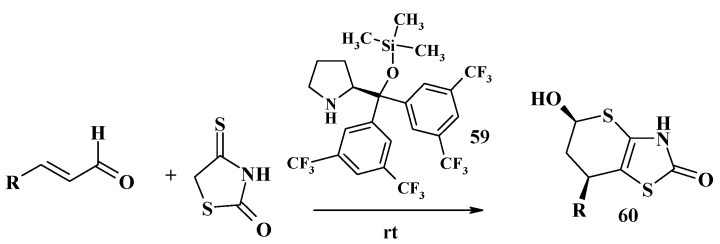
Divergent organocatalitic Michael-Michael-aldol cascade reaction in the synthesis of thiopyrano[2,3-*d*]thiazoles.

Unexpectedely, Zhang and coauthors obtained thiopyranoid scaffold **60** (Scheme 18) exploring the divergent organocatalitic Michael-Michael-aldol cascade reaction of isorhodanine with α,β-unsaturated aldehydes. Whilst the same conditions in the reaction of thiazolidinedione and rhodanine with enals led to spiro compounds, in the case of isorhodanine usage Michael cyclization took place. Optimizing the reaction conditions authors had used toluene medium and organic catalyst **59** at room temperature [44].

## 4. Synthesis of Polycondensed Thiopyrano[2,3-*d*]Thiazole Derivatives as Potentially Biological Active Compounds

The tandem and “domino” processes based on [4+2]-cycloaddition reaction is a powerful and effective tool in the synthesis of thiopyrano[2,3-*d*]thiazole derivatives. This type of reactions allows the synthesis of structurally complex molecules with high selectivity, while the consumption of solvents, reagents, adsorbents and energy is significantly reduced comparing with traditional multistage synthetic approaches. Moreover, most of the tandem and “domino” reactions products have drug-like structure and probably may possess interesting pharmacological effects that is important point in the modern process of drugs development. 

### 4.1. Peculiarities of the Tandem Reactions in the Synthesis of Polycondensed Thiopyrano[2,3-d]Thiazoles 

**Scheme 19:**
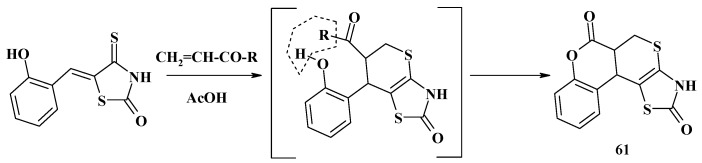
The first example of tandem *hetero*-Diels-Alder reaction for the 5-arylidene-4-thiazolidinethiones.

Presence of active groups in the *o*-position of arylidene fragment in 5-arylidene-4-thiazolidinethiones is an important feature contributing to the passing of tandem processes based on *hetero*-Diels–Alder and Michael reactions in the synthesis of thiopyrano[2,3-*d*]thiazoles. For example, I.D. Komaritsa [18] and N.A. Kassab [20] had used 5-(2-hydroxyphenylmethylidene)-isorhodanine in the reactions with acrylic acid, its ethyl ester or amide to obtaine 3,5a,6,11b-tetra-2*H*,5*H*-chromeno[4′,3′:4,5]thiopyrano[2,3-*d*]thiazole-2,6-dione with high yields **61**. Mentioned reaction is a two-step process involving combination of hetero diene synthesis and acylation of phenolic group and was the first example of tandem *hetero*-Diels–Alder reaction for the 5-arylidene-4-thiazolidinethiones (Scheme 19).

**Scheme 20:**
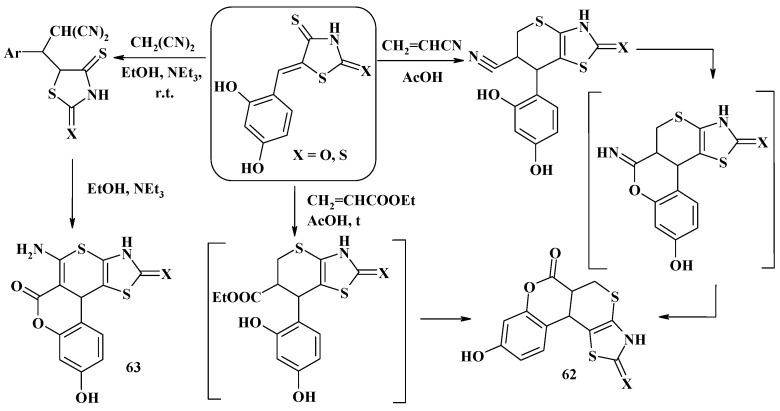
Tandem reaction of 5-(2,4-dihydroxyphenylmetylidene)iso(thio)rhodanines with ethyl acrylate and acrylonitrile.

Based on experimental conclusions of I.D. Komaritsa and N.A. Kassab, Metwally and coauthors [45] performed similar tandem process synthesizing a polycyclic system **62** in the reaction of 5-(2,4-dihydroxyphenylmethylidene)iso(thio)rhodanines with ethyl acrylate and acrylonitrile. Moreover, the Michael reaction with malononitrile led to the structurally similar 9-hydroxybenzo[3′,4′:4,5]thiopyrano[2,3-*d*]thiazol-6-one **63** (Scheme 20).

**Scheme 21:**
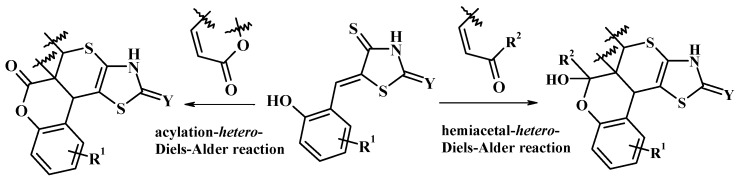
Two types of 5-(2-hydroxyphenylmethylidene)-4-thiazolidinethione-based tandem *hetero*-Diels-Alder reactions.

Among the tandem *hetero*-Diels–Alder reactions, two types of processes can be distinguished: acylation- and hemiacetal-based reactions (Scheme 21). The first approach requires the usage of derivatives of α,β-unsaturated carboxylic acids as dienophiles, and the second—α,β-unsaturated oxo compounds (aldehydes and ketones). 

**Scheme 22:**
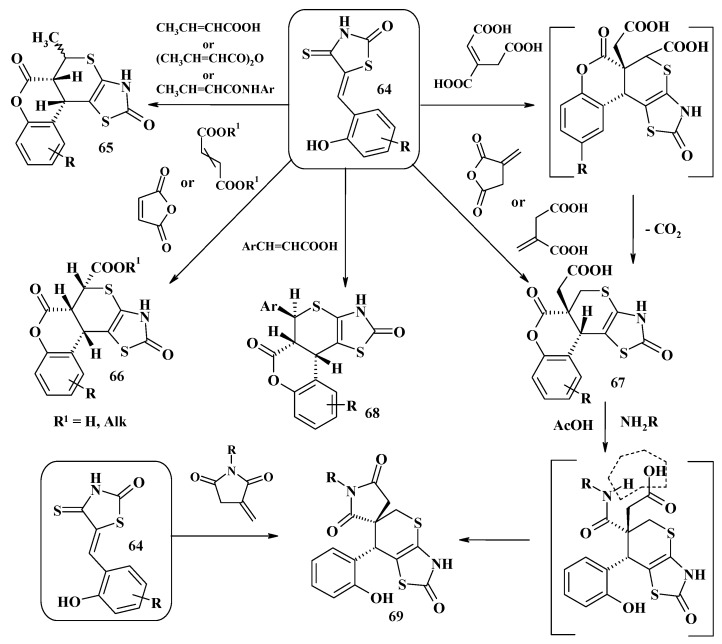
Crotonic, cynnamic, propiolic, maleic, fumaric, itaconic and *trans*-aconitic acids motifs in the synthesis of thiopyrano[2,3-*d*]thiazoles via tandem acylation *hetero*-Diels–Alder reaction.

Thus, when studying *hetero*-Diels–Alder-acylation tandem reactions of 5-(2-hydroxyphenylmethylidene)isorhodanines **64** with unsaturated carboxylic acids and their derivatives more precisely, a number of stereochemical peculiarities of these processes were established (Scheme 22). For example, in the reaction of crotonic acid, its amides or anhydride a mixture of *rel*-5*R*,5a*R*,11b*S* and *rel*-5*S*,5a*R*,11b*S* diastereomers (**65**) were formed; the isomers’ ratio depends on the nature of dienophile and the reaction temperature [26]. The reaction of heterodiene **64** with maleic and fumaric acids and their derivatives (maleic anhydride, esters) passed diastereoselectively [46,47]. Moreover, independently of the stereoisomerism of the dienophile a racemic mixture of *rel*-(5*R*,5a*R*,11b*S*)-2,6-dioxo-3,5a,6,11b-tetrahydro-2*H*,5*H*-chromeno[4′,3′:4,5] thiopyrano[2,3-*d*]thiazole-5-carboxylic acids derivatives **66** with a cis-Hydrogen in positions 5, 5α, and 11β of heterocyclic systems was formed. Itaconic acid and its anhydride [48,49] as well as *trans*-aconitic acid [33] reacted in a similar manner forming derivative **67**. In the case of *trans*-aconitic acid the reaction proceeded with spontaneous decarboxylation at position 5 of thiopyrano[2,3-*d*]thiazole core [33]. *rel*-(5*S*,5a*R*,11b*S*)-5-Aryl-3,5a,6,11b-tetrahydro-2*H*,5*H*-chromeno [4′,3′:4,5]thiopyrano[2,3-*d*]thiazole-2,6-diones **68** were the products of tandem *hetero*-Diels–Alder-acylation reaction of 5-(2-hydroxyphenylmethylidene)isorhodanines and cynnamic acids [26]. Compound **67** proved to be effective reagent for the next chemical transformations. The reaction of **67** with primary amines in acetic acid passed through the amidation stage, followed by spontaneous recycling in spiroimides **68**. The thiopyrano[2,3-*d*]thiazoles **68** were also obtained by the alternative method from itaconic acid imides [48].

**Scheme 23:**
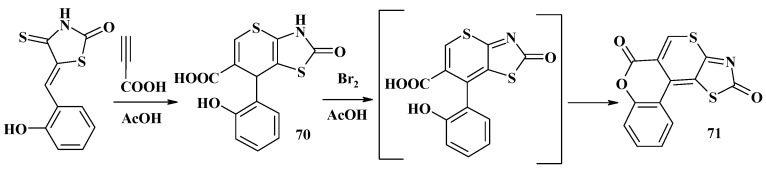
Synthesis of 2*H*,6*H*-chromeno[4′,3′:4,5]thiopyrano[2,3-*d*]thiazole-2,6-dione.

It is important to note that at interaction of 5-(2-hydroxyphenylmetylidene)isorhodanine with propiolic acid, a classic *hetero*-Diels–Alder reaction takes place to form thiopyrano[2,3-*d*]thiazole derivative **70**. The presence of a double bond at positions 5–6 causes planar structure of the bicyclic fragment and creates the spatial obstacles for acylation of phenolic group. Dehydrogenation of basic heterocycle with bromine in acetic acid removes these obstacles and allows obtaining tetracyclic 2*H*,6*H*-chromeno[4′,3′:4,5]thiopyrano[2,3-*d*]thiazole-2,6-dione **71** (Scheme 23) [26].

**Scheme 24:**
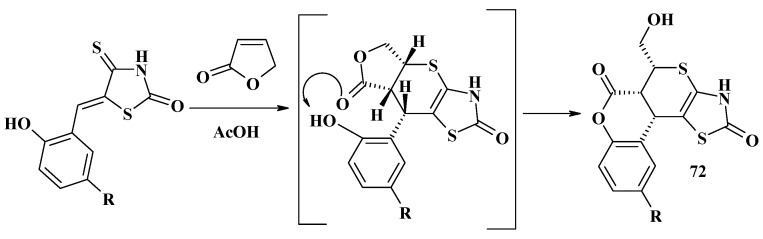
2(5*H*)Furanone motif in the synthesis of thiopyrano[2,3-*d*]thiazoles via tandem acylation *hetero*-Diels-Alder reaction.

The reaction of 5-(2-hydroxyphenylmethylidene)isorhodanines with 2(5*H*)furanone proceeded as a diastereoselective tandem acylation-*hetero*-Diels-Alder reaction providing novel *rel*-(5*R*,5a*R*,11b*S*)-5-hydroxymethyl-3,5,5a,11b-tetrahydro-2*H*,5*H*-chromeno[4′,3′:4,5]thiopyrano[2,3-*d*]thiazole-2,6-diones **72** (Scheme 24) [32].

**Scheme 25:**
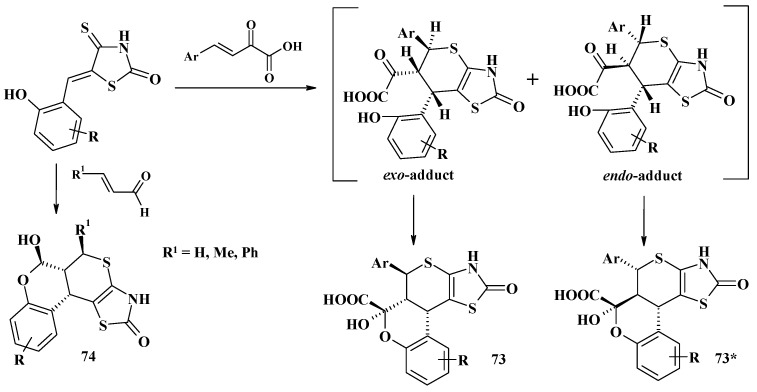
Features of tandem *hetero*-Diels-Alder-hemiacetal reactions.

The reactions between 5-(2-hydroxybenzylidene)-4-thioxo-2-thiazolidinones and arylidene pyruvic acids yielded the mixture of *rel*-(5*S*,5a*R*,11b*R*) **73** and *rel*-(5*R*,5a*S*,11b*R*) 73* diastereisomers at 2:1 ratio [50]. At the same time acroleine, crotonic and cynnamic aldehydes in mentioned tandem *hetero*-Diels-Alder-hemiacetal reaction (Scheme 25) diastereoselectively yielded novel *rel*-(5a*R*,6*R*,11b*S*)-6-hydroxy-3,5a,6,11b-tetrahydro-2*H*,5*H*-chromeno[4′,3′:4,5]thiopyrano[2,3-*d*]thiazole-2-ones **74** [51].

### 4.2. Domino Reactions as a Systematic Approach to the Synthesis of Fused Thiopyrano[2,3-d]Thiazoles

In addition to tandem reactions, domino reactions also play an important role in the synthesis of thiopyrano[2,3-*d*]thiazoles of complex structure. A domino reaction involves two or more transformations, which result in the formation of bonds (usually C–C bonds) and occur under the same reaction conditions without adding new reagents and/or catalysts. In this process the subsequent reactions take place as a consequence of the functionality formed in the previous step [52].

**Scheme 26:**
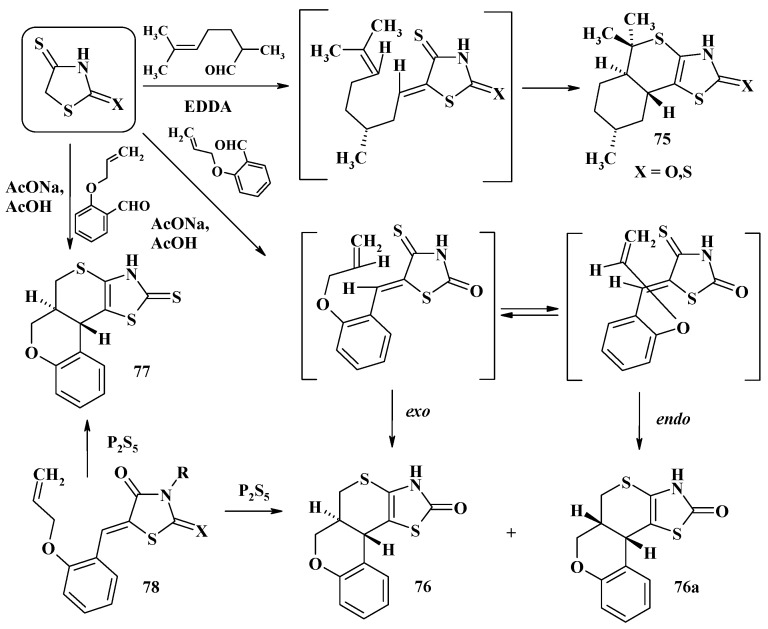
Features of domino Knoevenagel-*hetero*-Diels–Alder reaction.

So, one of the examples of thiopyranothiazole scaffold synthesis is obtaining of series of chromeno[4′,3′:4,5]thiopyrano[2,3-*d*]thiazole-2-(thi)ones **76**,**77** and isothiochromeno[4a,4-*d*]thiazole-2-ones **75** in the domino Knoevenagel-*hetero*-Diels–Alder reaction (Scheme 26) of isorhodanine with 3,7-dimethyl-6-octenal ((±)citronelal) and 2-allyloxybenzaldehyde. It should be noted that the reaction of isorhodanine with 2-allyloxybenzaldehyde yielded a mixture of trans-**76** and cis-**76a** isomers (5:1). The authors proposed a probable mechanism of this reaction through the exo-selective cyclization of the 5-arylidene intermediate in *hetero*-Diels-Alder reaction, while the formation of endo-intermediate is a minor process. Recrystallization from dioxane can provide individual trans isomer **76** [53]. Tetracyclic derivatives **76**,**77** were synthesized alternatively via the “domino” thionation-*hetero*-Diels-Alder reaction of 5-(2-allyloxyphenylmethylidene)-4-thiazolidinones **78** [54].

**Scheme 27:**
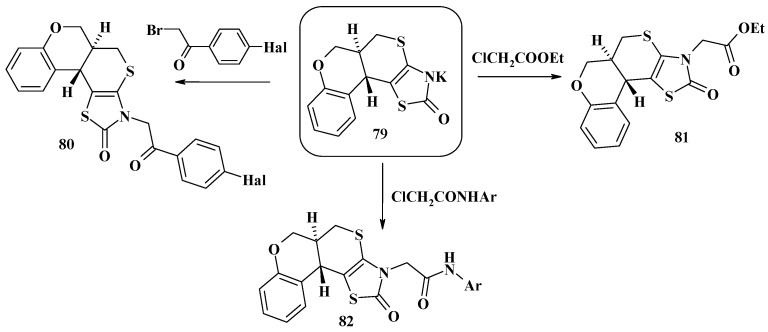
Synthesis of 3-substituted chromeno[4′,3′:4,5]thiopyrano[2,3-*d*]thiazole-2-ones.

The NH-acidic center of the basic core of the compound **76** predetermined its further transformations (Scheme 27) through the stage of potassium salt **79** formation with the following alkylation with chloroacetamides, bromoacetophenones, ethyl chloroacetate that allowed obtaining new *N*-substituted derivatives **80**–**82** [55].

**Scheme 28:**
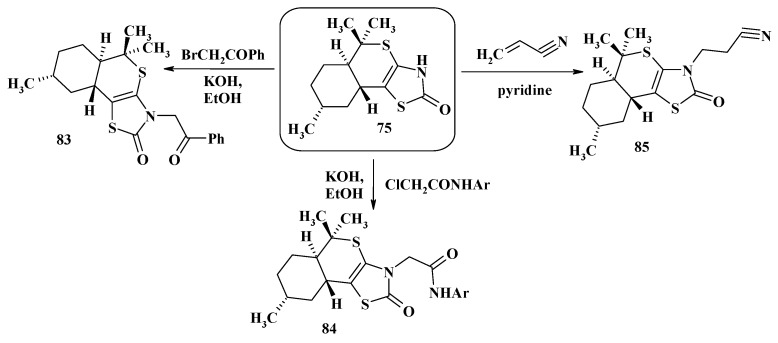
Synthesis of 3-substituted isothiochromeno[4*a*,4-*d*]thiazole-2-ones.

Using the same approach (Scheme 28), isothiochromeno[4a,4-*d*]thiazole-2-one **75** was alkylated by chloroacetamides, bromoacetophenones to give the derivatives **83**,**84**. When it was treated with acrylonitrile, the cyanoethylation reaction resulted in propionitryl **85** formation [56].

**Scheme 29:**
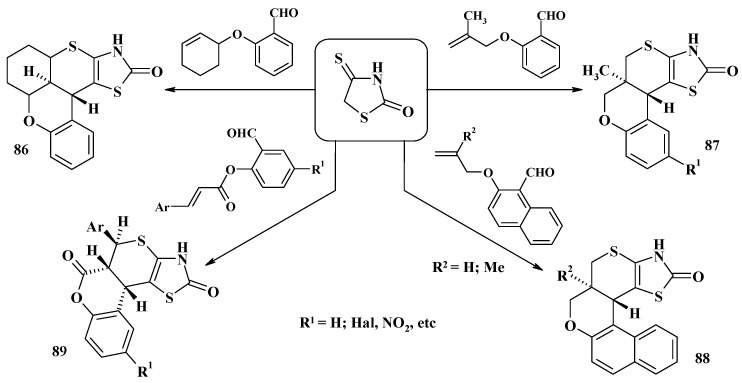
Domino Knoevenagel-*hetero*-Diels-Alder reaction of isorhodanine with 2-(2-methylallyloxy)- and 2-(cyclohexene-2-yloxy)benzaldehydes, 2-allyloxynaphthalaldehyde and 2-formylphenyl-(*E*)-3-aryl-2-propenoates.

Another example of the domino Knoevenagel-*hetero*-Diels-Alder reaction (Scheme 29) is the interaction of isorhodanine with structural analogs of 2-allyloxybenzaldehyde-2-(2-methylallyloxy)- and 2-(cyclohexene-2-yloxy)benzaldehydes, 2-allyloxynaphthalaldehyde as well as 2-formylphenyl-(*E*)-3-aryl-2-propenoates. These reactions allowed preparing a series of pentacyclic derivatives characterized by trans-(**86**–**88**) or cis-configuration (**89**) of 5a and 11b protons [53]. Interestingly, that when 2-formylphenyl-(*E*)-3-aryl-2-propenoates are used as reagents stereoconfiguration of final products **89** were similar to the derivatives **68** obtained in tandem acylation*-hetero*-Diels–Alder reaction (Scheme 22). Stereochemistry of final compounds depends on the endo- and exo-orientation of the dienophile in transition state. The presence of allyl moiety in the molecule induces exo-transition state, in contrast to cinnamoyl fragment which causes endo-orientation of the dienophile due to the orbital interactions.

**Scheme 30:**
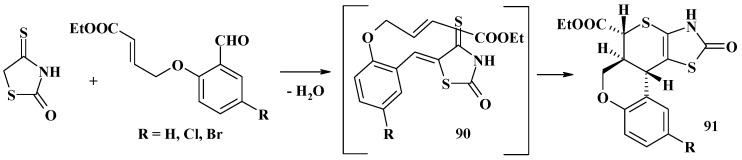
Domino Knoevenagel-*hetero*-Diels-Alder reaction between 4-thioxo-2-thiazolidinone and ethyl (2*E*)-4-(2-formylphenoxy)but-2-enoate.

The intramolecular Knoevenagel condensation (Scheme 30) between the ethyl (2*E*)-4-(2-formylphenoxy)but-2-enoate and 4-thioxo-2-thiazolidinone affords intermediate **90** for intramolecular *hetero*-Diels–Alder cycloaddition providing diastereoselective formation of ethyl *rel*-(5a*R*,5*R*,11b*R*)-2-oxo-2,3,5,5a,6,11b-hexahydrochromeno[4′,3′:4,5]thiopyrano[2,3-*d*]thiazole-5-carboxylates **91** [46].

**Scheme 31:**
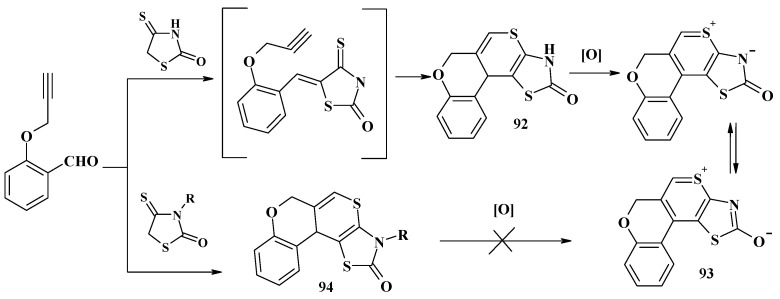
Peculiarities of 2-(2-propynoxy)benzaldehyde in the domino reactions with isorhodanine.

The reaction of isorhodanine with 2-(2-propynoxy)benzaldehyde in the medium of acetic acid in the presence of catalytic amount of sodium acetate did not stop on the formation of expected 3,11b-dihydro-2*H*,6*H*-chromeno[4′,3′:4,5]thiopyrano[2,3-*d*]thiazole-2-one **92**. The latter underwent spontaneous oxidation yielding 6*H*-chromeno[4′,3′:4,5]thiopyrano[2,3-*d*]thiazole-4-ium-2-olate **93**. *N*-substituted isorhodanines traditionally reacted in the domino Knoevenagel-*hetero*-Diels–Alder reaction providing derivatives **94** (Scheme 31) [57].

## 5. Biological Activity of Thiopyrano[2,3-*d*]Thiazole Derivatives

One of the efficient and freguently used directions of search for new active compounds is based on the principle of privileged structures annealing in the condensed systems. This approach involves combination of different heterocyclic pharmacophores in one molecule and can be successfully illustrated by thiopyrano[2,3-*d*]thiazoles. Taking into account that thiopyrano[2,3-*d*]thiazole derivatives are cyclic isosteric mimetics of 5-ene-4-thiazolidinones without typical Michael acceptors properties, the study of possible biological activity of these compounds is of great interest.

**Figure 3:**
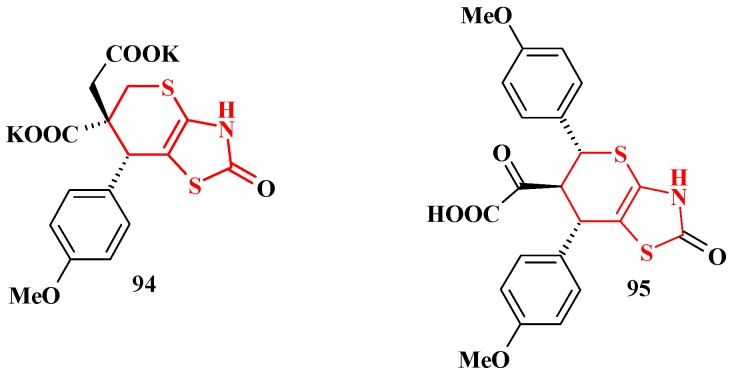
Thiopyrano[2,3-*d*]thiazoles as potenstial NSAIDs.

Thiopyrano[2,3-*d*]thiazoles were studied as potential non-sterodial anti-inflammatory drugs (NSAIDs). For example, anti-inflammatory activity (Figure 3) of 6-carboxymethyl-7-(4-methoxyphenyl)-2-oxo-3,5,6,7-tetrahydro-2*H*-thiopyrano[2,3-*d*]thiazole-6-carboxylic acid potassium salt **94** identified in the carrageenan-induced paw edema model in the rats, was comparable with such showed by the reference drugs diclofenac sodium [58]. A similar activity level was established for the *rel*-(5*R*,6*S*,7*S*)-[5-(3,4-dimethoxyphenyl)-7-(4-methoxyphenyl)-2-oxo-3,5,6,7-tetrahydro-2*H*-thiopyrano[2,3-*d*]thiazol-6-yl]-oxoacetic acid **95** [29].

**Figure 4:**
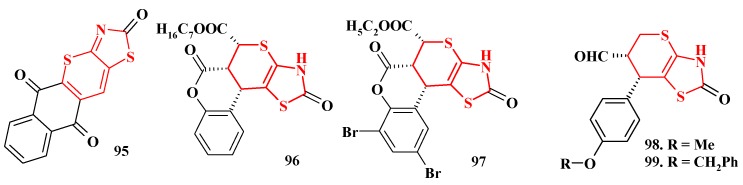
Thiopyrano[2,3-*d*]thiazoles with antiviral activity.

Like many other classes of synthetic small molecules, a group of 4-thiazolidinone-based derivatives was used in the search for novel selective antiviral agents [59,60]. 5,10-dihydro-*2H*-benzo[6,7]thiochromeno[2,3-*d*]thiazol-2,5,10-trione **96** possessed moderate activity against coronavirus SARS (EC_50_ = 1.7 μM, SI = 14). The above-mentioned derivative had also showed micromolar ranges of cancer cell lines inhibition (GI_50_ = 1.95 μM) and low toxicity levels (IC_50_ = 23 μM), being the most active towards some lines of leukemia, non-small cell lung cancer and melanoma [40]. One more class of thiopyranothiazole derivatives being tested for antiviral activity was chromeno[4′,3′:4,5]thiopyrano[2,3-*d*]thiazoles **97**,**98** which showed inhibition activity against influenza virus type A H_3_N_2_ and H_5_N_1_ (Figure 4) [47,58]. 7-Aryl-2-oxo-3,5,6,7-tetrahydro-2*H*-thiopyrano[2,3-*d*]thiazole-6-carbaldehydes possesed promising influence on EBV virus (compound **98**, EC_50_ = 0.07 μM, SI = 3279) and Hepatitis C virus (compound **99**, EC_50_ = 12.6 μM, SI = 43.1), respectively [31].

**Figure 5:**
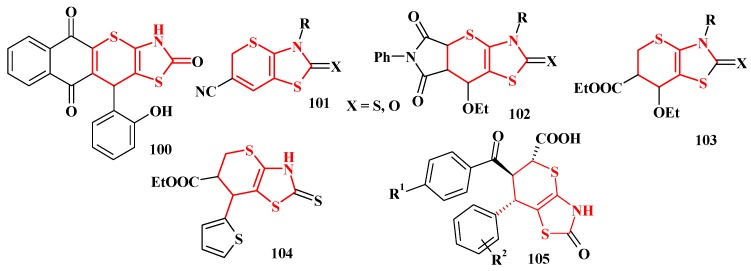
Thiopyrano[2,3-*d*]thiazoles with antimicrobial, antifungal and antimycobacterial activities.

Study of antituberculosis activity of thiopyranothiazoles allowed identifying a hit-compound 11-(2-hydroxyphenyl)-3,11-dihydro-2*H*-benzo[6,7]thiochromeno[2,3-*d*]thiazol-2,5,10-trione **100** with promising antimycobacterial activity (MIC_90_ 0.6 µg/mL, *Mycobacterium tuberculosis* H_37_R_v_ strain) and low in vivo toxicity profile [17]. In general, antimicrobial, antifungal and antimycobacterial profiles were among the first types of biological activities described for the thiopyranothiazoles (Figure 5). In particular, in the screening of structurally simple thiopyrano[2,3-*d*]thiazoles using the disk-diffusion method moderate/high antimicrobial and antifungal activities against *Pseudomonas lachrymans*, *Erwinia toxica*, *Penicillium simplex*, *Ulocladium chertorium* and *Aspergillus oryzae* were identified for the derivatives **101** and **102**. Moreover, derivatives of the structure **103** inhibited growth of *Mycobacterium sp.* [39]*.* Study of a series of 5-(2-thienyl)methylene-4-thioxo-2-thiazolidinones and their condensed analogs resulted in identifying of potent active compound 6-carboxy-7-(2-thienyl)-tetrahydrothiopyrano-7*H*-[2,3-*d*]thiazole **104**, which had shown besides antimicrobial (against *B. Subtilis*) and antituberculous activities high antifungal potential (Figure 5) [61,62]. Significant influence on *Staphylococcus aureus*, *Bacillus subtilis* and *Candida albicans* was identified for the *rel-*(5*R*,6*S*,7*R*)-6-benzoyl-7-phenyl-2-oxo-3,5,6,7-tetrahydro-2*H*-thiopyrano[2,3-*d*] thiazole-5-carboxylic acids **105** [28].

**Figure 6:**
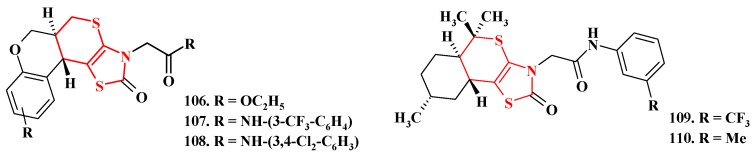
Chromeno[4′,3′:4,5]thiopyrano[2,3-*d*]thiazoles and isothiochromeno[3,4-*d*]thiazoles with anticancer activity in vitro.

One of the most popular and promising directions of the fused 4-thiazolidinone derivatives investigation is the search for anticancer agents. However, unlike monocyclic 4-thiazolidinones, the mechanism of their antitumor activity is not well understood yet. Moreover, quite often, biophore fragments of the active 4-thiazolidinone derivatives save their pharmacological effect in the new polycyclic scaffold. The latter may be considered as an argument in favour of the given class of compounds as a source of «small drug-like molecules», in particular anticancer agents. Thus, among fused polycyclic thiazolothiopyranes a large number of compounds characterized by the high inhibition rates and/or cytostatic activity towards cell lines of leukemia, non-small cell lung cancer, renal cancer, melanoma, ovarian cancer, breast cancer, prostate and CNS cancer were identified. A series of *N*-substituted chromeno[4′,3′:4,5]thiopyrano[2,3-*d*]thiazoles revealed sufficiently high level of growth inhibition of different cancer cells with the avarage logGI_50_ values from −4.49 to −6.22 in the in vitro studies (Figure 6). The most active were compounds with the ester-**106**, *N*-trifluoromethylphenyl- and 3,4-dichlorophenyl-acetamide fragments (**107**,**108**) in the molecules that were characterized by the cytostatic effects against a panel of cancer cell lines. On the other hand, 4-methylphenylacetamide substituent in the N3 position of tetracycle increased its selective activity against leukemia cell lines, which was observed at submicromolar concentrations (logGI_50_ = −6.93 for *CCRF-CEM* and < −8.00 for *HL-60(TB)*), whilst its inhibition activity against other cancer cells turned out to be relatively low [55]. Interestingly that structurally related fused system of isothiochromeno[3,4-*d*]thiazole (Figure 6) with 3-trifluoromethylphenylacetamide **109** and 3-methylphenylacetamide **110** substituents in the N3 position had also showed high antitumor effects along with low acute cytotoxicity levels [54,56].

**Figure 7:**
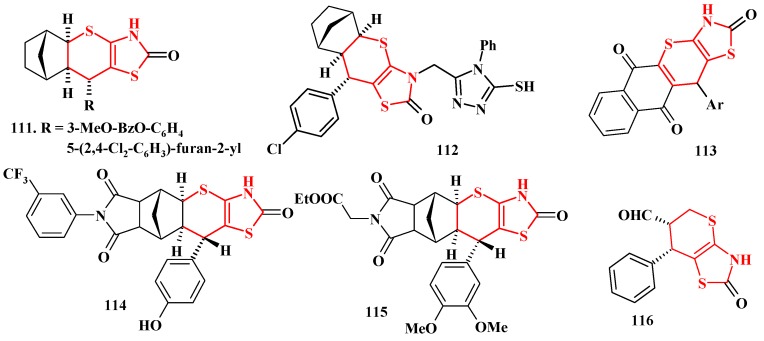
Thiopyrano[2,3-*d*]thiazoles with anticancer activity in vitro.

One of the chemical modification directions of thiopyranothiazoles is the introduction of norbornane fragment in the molecules (Figure 7). Thus, thopyranothiazole systems of type **111** with 4-benzyloxy-3-methoxyphenyl- and 5-(2,5-dichlorophenyl)-furan-2-yl substituents in the 9th position of the main core inhibited cancer cell growth at submicromolar concentrations [15]. Structurally related *N*-substituted 9-aryl(heteryl)-3,7-dithia-5-azatetracyclo[9.2.1.0^2,10^.0^4,8^] tetradecen-4(8)-one-6 **112** being moderately active towards a panel of cancer cell lines, showed rather good growth inhibition against leukemia cell lines [63]. Further modification of the main thiopyranothiazole scaffold allowed synthesis of the derivatives with naphthoquinone fragment **113**. The latter showed high level of anticancer activity with moderate selectivity towards melanoma cells. It is worth mentioning that 11-substituted benzo[6,7]thiochromeno[2,3-*d*]thiazole-2,5,10-triones had also antituberculosis activity (compound **98**, Figure 5) and low acute toxicity [17]. High anticancer activity was identified in the row of 3,7-dithia-5,14-diazapenthacyclo[9.5.1.0^2,10^.0^4,8.^0^12,16^]heptadecenes. The most active were the hit-compounds **114** and **115**, herewith **114** selectively inhibited growth of leukemia cell lines CCRF-CEM (Log GI_50_ = −6.40) and SR (Log GI_50_ = −6.06) [16]. 7-Phenyl-2-oxo-7-phenyl-3,5,6,7-tetrahydro-2*H*-thiopyrano[2,3-*d*]thiazole-6-carbaldehyde **116** showed high level of antimitotic activity against leukemia with mean GI_50_/TGI values 1.26/25.22 μM [31].

**Figure 8:**
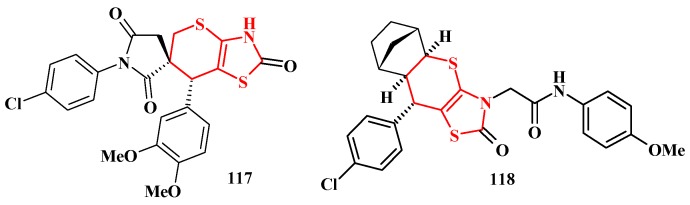
Thiopyrano[2,3-*d*]thiazoles with trypanocidal activity.

One of the promising and quite new directions of thiazolidinone derivative investigations is the search for potent anti-parasitic agents, namely compounds exhibiting antitrypanosomal activity. Trypanosomiasis belongs to the so called world’s neglected diseases caused by *Trypanosoma spp*. [64]. Among spiro thiopyrano[2,3-*d*]thiazole **117** derivatives an active compound inhibiting growth of *Trypanosoma brucei brucei* and *Trypanosoma brucei gambiense* (the causative agent of African trypanosomiasis) with the IC_50_ values of 0.26 µM and 0.42 µM, respectively, was identified [48]. Interesting is dual anti-leukemic (log GI_50_ = −5.16, −5.59) and trypanocidal effects observed for thiopyranothiazole **118** bearing norbornane moiety that may be used for establishing molecular modes of action for this class of compounds (Figure 8) [63].

**Figure 9:**
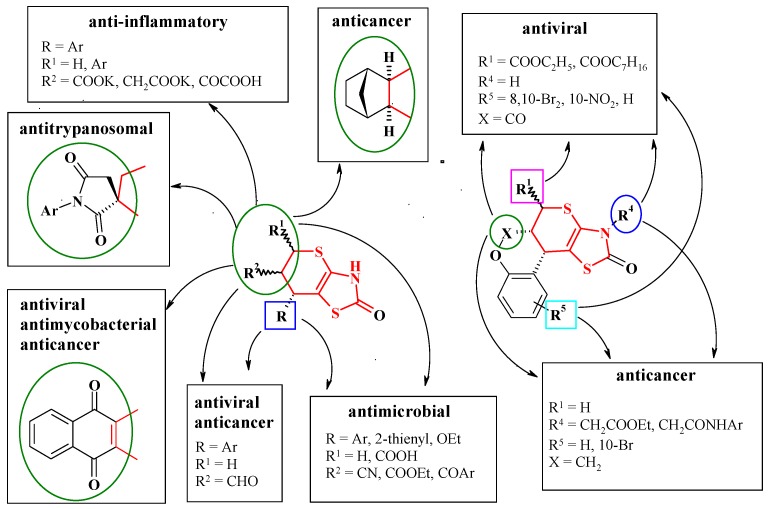
Some structure-activity relationships of thiopyrano[2,3-*d*]thiazoles.

Summarising all the above, fused thiopyranothiazoles can be used as a source for new antibacterial as well as antiviral agents. They also inhibited parasites growth. These results correlate with established anticancer profiles of the thiopyranothiazoles. Moreover, such fused heterocycles can be investigated as potent non-steroidal anti-inflammatory agents. Some structure-activity relationships are outlined in the Figure 9.

## 6. Conclusions

The efficient approaches to the thiopyranothiazoles scaffolds synthesis are outlined in this review. One of the most studied synthetic protocol for thiopyranothiazoles is the *hetero*-Diels–Alder [4+2]-cycloaddition being rather fast and efficient method that yields good outcomes and stereoselectivity of the products. The tandem processes based on *hetero*-Diels–Alder and Michael reactions used for the thiopirano[2,3-*d*]thiazoles synthesis have also been discussed. In contrast to the well discribed various synthetic routes of thiopyranothiazoles synthesis, biological activity of these derivatives have not been studied that much. Nevertheless, they are considered as 5-ene-4-thiazolidinone synthetic biomimetics that save pharmacological profile without revealing Michael acceptors properties. Among established biological activities of the thiopyrano[2,3-*d*]thiazole derivatives, the anti-inflammatory, antibacterial, anticancer as well as aniparasitic activities are the most prominent and need further in-depth studies. Considering all the above, the directed search for new drug-like molecules and possible chemotherapeutic agents among thiopyrano[2,3-*d*]thiazole derivatives is justified and promising direction in the medicinal chemistry. Moreover, the way of annealing of thiazolidine core into thiopyranothiazole analogs is used as one of the molecular optimization directions to decrease the toxicity and/or avoid the Michael acceptor properties as well.
